# Supplementary material for: Searching for an exotic spin-dependent interaction with a single electron-spin quantum sensor
Source: Nat Commun. 2018 Feb 21;9:739. doi: 10.1038/s41467-018-03152-9 (PMC5821819; doi:10.1038/s41467-018-03152-9)
Supplement: Supplementary file 1 — Supplementary Information [file 41467_2018_3152_MOESM1_ESM.pdf]

Supplementary Information for ”Searching for an exotic spin-dependent interaction with a single electron-spin quantum sensor”

Rong *et al.*

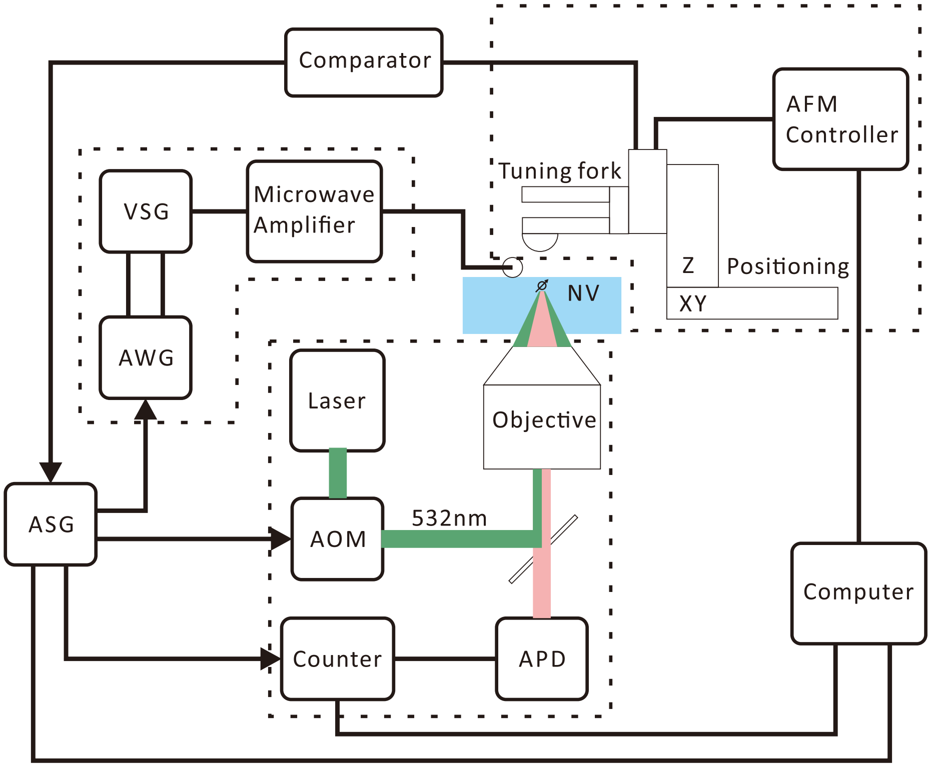

Supplementary Figure 1: **Schematic of the experimental setup.** An optically detected magnetic resonance (ODMR) setup, which consists of an optical system to initialize and read out the electron spin state of the nitrogen-vacancy (NV) center and a microwave system to manipulate the electron spin state of NV center, and a tuning fork based atomic force microscope to position and drive the half-ball lens, are synchronized by an arbitrary sequence generator (ASG).

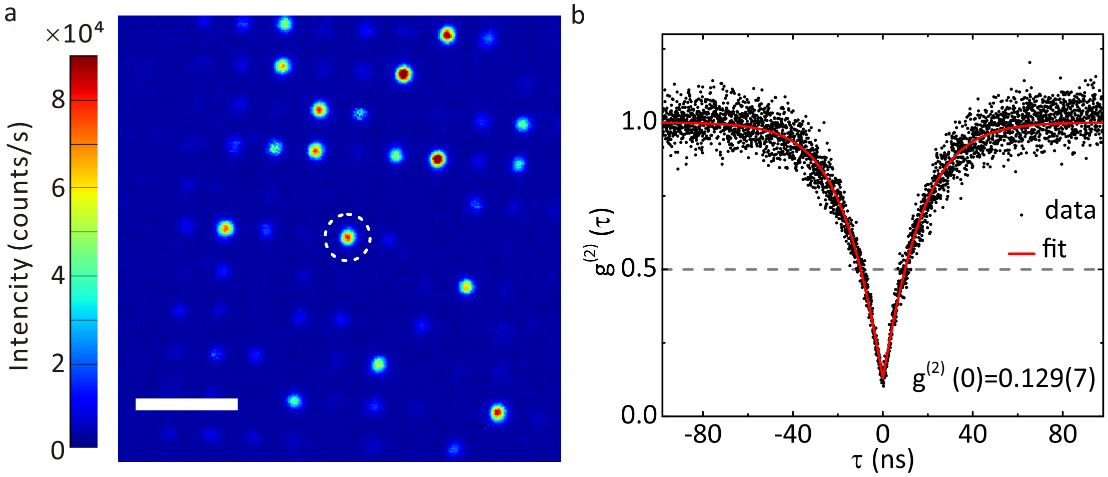

Supplementary Figure 2: **NV center in this experiment.** (**a**) Confocal image of the NV centers. The length of the scale bar corresponds to $5\ \mu$m. The dashed white circle shows the NV center used in this experiment. (**b**) Measurement of the second-order correlation function $g^{(2)}(\tau)$ of the NV center in this experiment with $g^{(2)}(0) = 0.129\,(7)$. The grey dashed line indicates $g^{(2)}(\tau) = 0.5$. Black points indicate data (without background subtraction), and the red line is a fit to the function $g^{(2)}(\tau) = 1 + \frac{1}{N}\left(C_1 \cdot e^{-|\tau/\tau_1|} + C_2 \cdot e^{-|\tau/\tau_2|}\right)$.

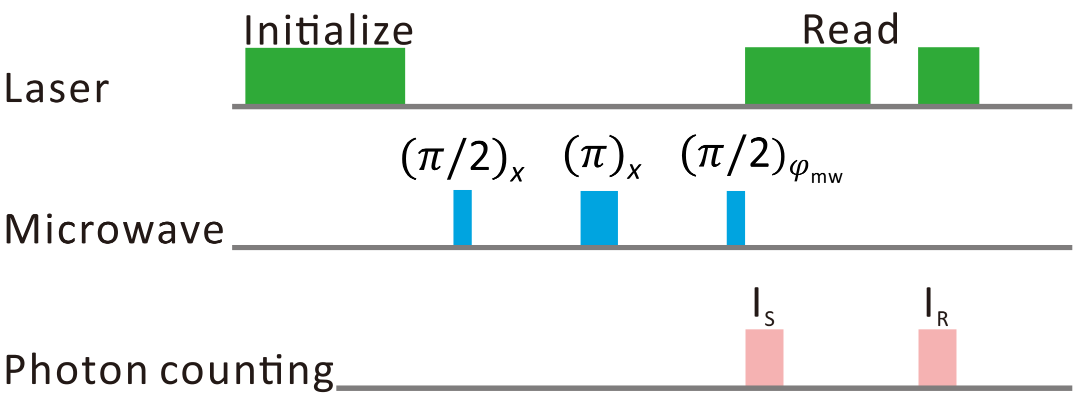

Supplementary Figure 3: **Pulse sequence of the experiment.**

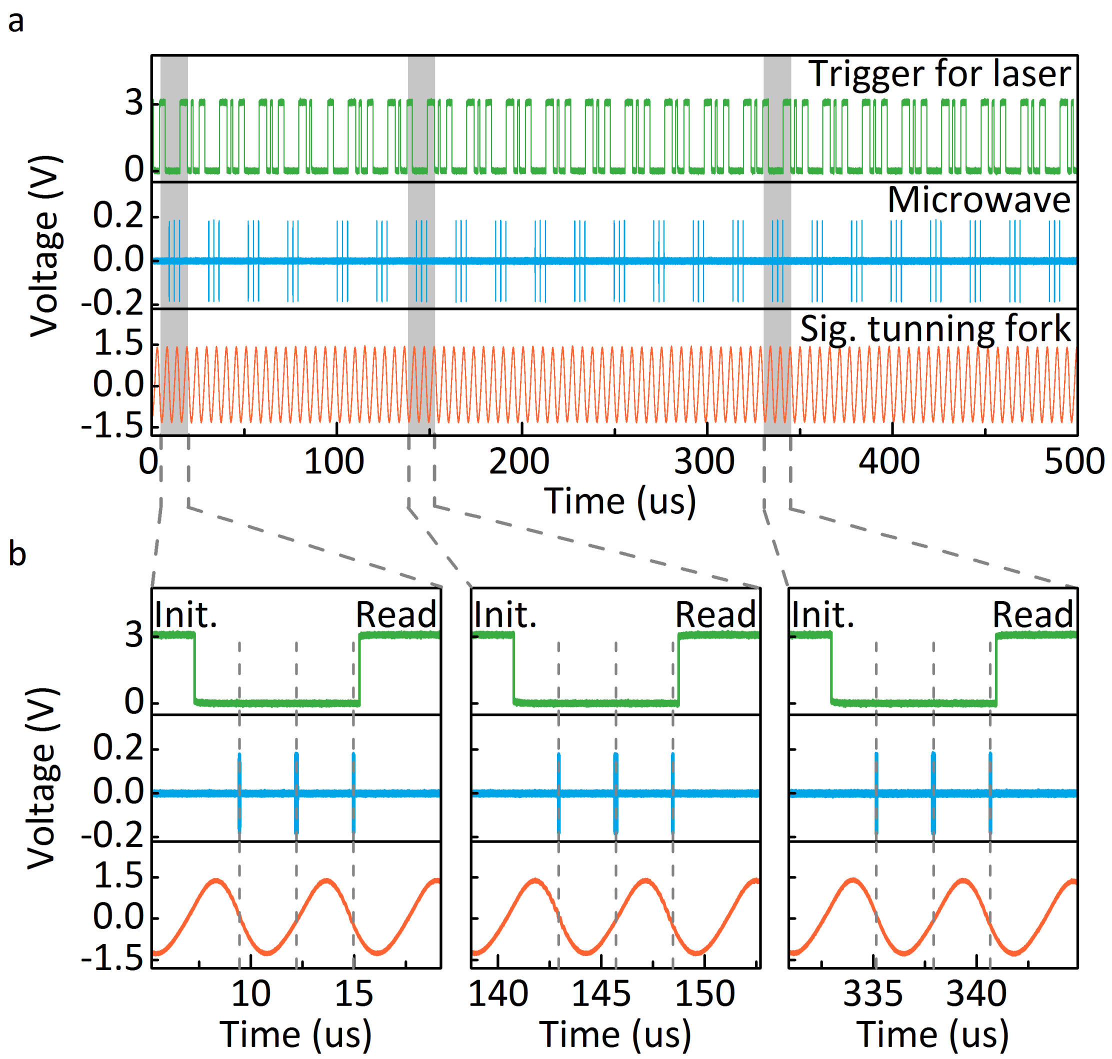

Supplementary Figure 4: **Experimental time sequence.** (**a**) Experimental sequence recorded in 500 μs time scale. The green line is the TTL trigger signal for laser pulses. The blue line is the experimental microwave pulses. The red line is the electronic signal from the tuning fork. (**b**) Magnification of the sequence showing the synchronization.

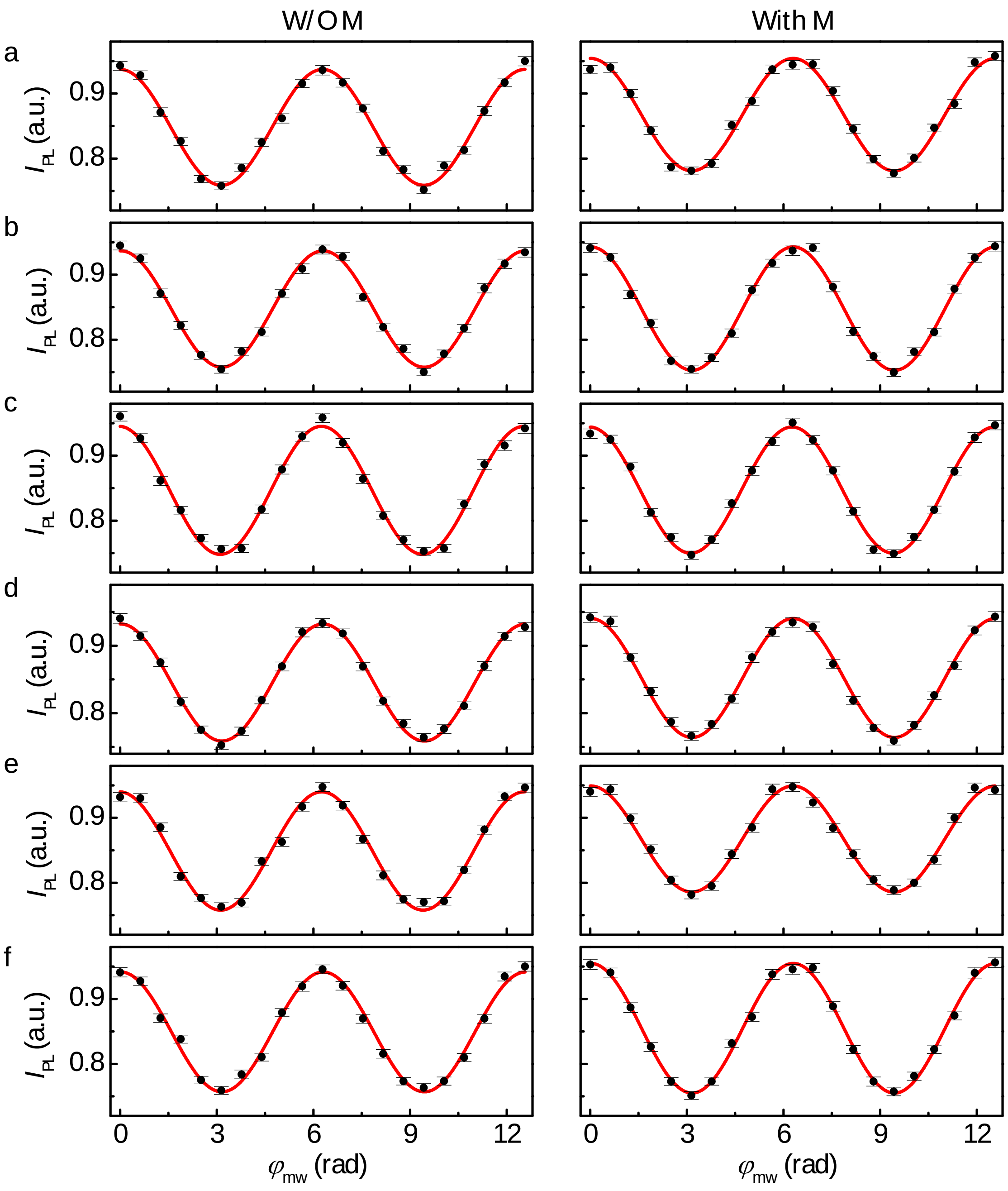

Supplementary Figure 5: **Six separated experimental runs.** Each experimental run contains one million trails. Left panels in (**a-f**) are six measurements without mass. Right panels in (**a-f**) are six measurements with mass. In both panels, the experimental data are represented by black circles with error bars, and the red solid lines represent the fitting of the experimental data. Error bars of the experimental data represent s.e.m.

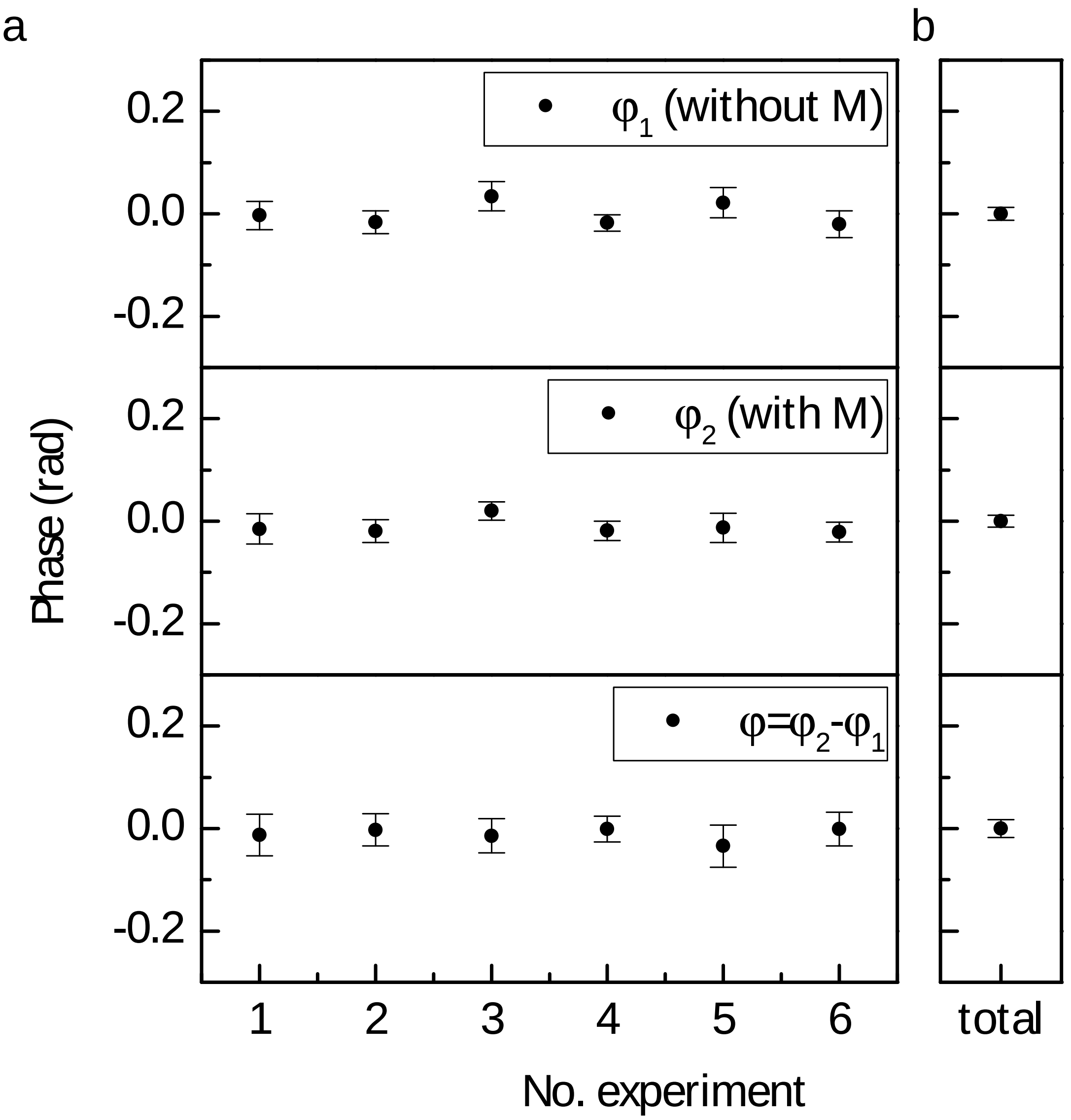

Supplementary Figure 6: **Phase differences between experiments with and without the mass.** (**a**) The obtained phases φ_1 and φ_2 are the accumulated phases of the states of S without and with M in six separated experimental runs, of which each run contains one million trails. The phase differences between experiments with and without the mass are presented in the lowest panel. The x axis stands for the xth experiments. (**b**) The accumulated phases, φ_1 and φ_2, are obtained if all the six experimental runs are considered. The phase difference are also obtained. In both subfigures, the error bars on φ_1 and φ_2 are fitting errors, and the error bars on φ are calculated according to the formula for propagation of error with errors on φ_1 and φ_2.

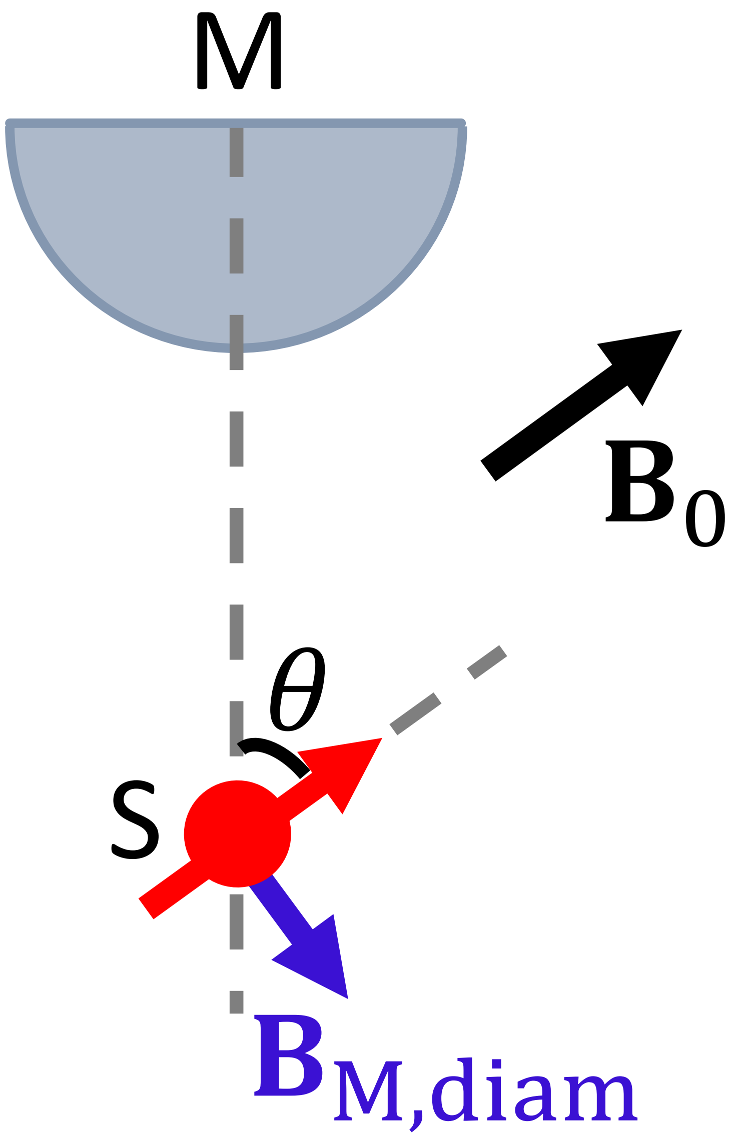

Supplementary Figure 7: **Schematic of the magnetic field on the NV center caused by the diamagnetism of the half-ball lens.** The red point with arrow labeled by S stands for the electron spin of the NV center. The semicircle labeled by M stands for the half-ball lens. The external magnetic field $\mathbf{B}_0$, which is applied along the NV symmetry axis, is presented with a black arrow. The angle θ between the NV symmetry axis and the symmetry axis of the half-ball lens is shown. The magnetic field on the NV center caused by the magnetism of the half-ball lens, which is denoted by $\mathbf{B}_{\mathrm{M,diam}}$, is schematically shown with a blue arrow. If the NV center locates exactly under the center of the half-ball lens, calculations show that $\mathbf{B}_{\mathrm{M,diam}}$ is perpendicular to the NV symmetry axis.

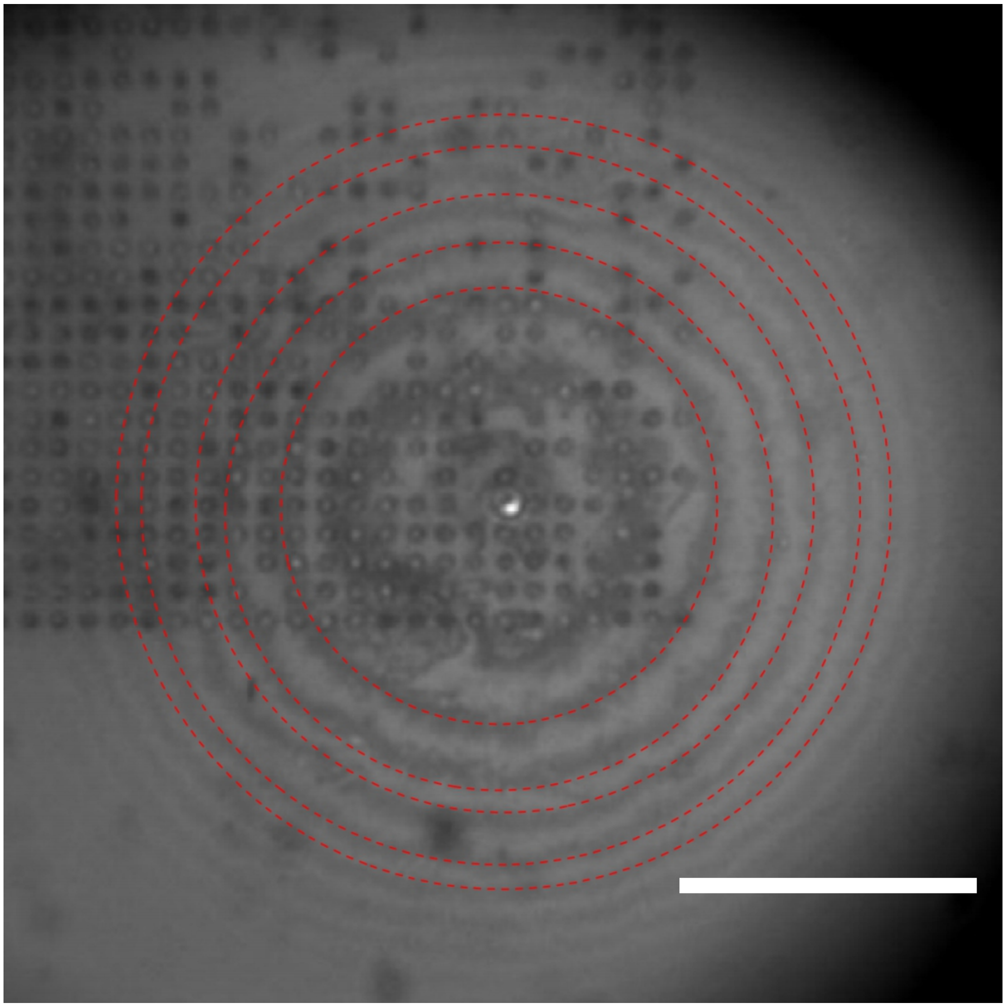

Supplementary Figure 8: **CCD image for estimation of the misalignment between the NV center and the half-ball lens.** The length of the scale bar corresponds to $20\ \mu$m. The light spot in the image shows the location of the NV center, which is focused in the laser light. The rings are Newton rings due to the half-ball lens. The dashed lines are fitting results to the positions of Newton rings. The misalignment between the NV center and the half-ball lens is estimated to be $0.7(8)\ \mu$m according to the location of the NV center and the center of Newton rings.

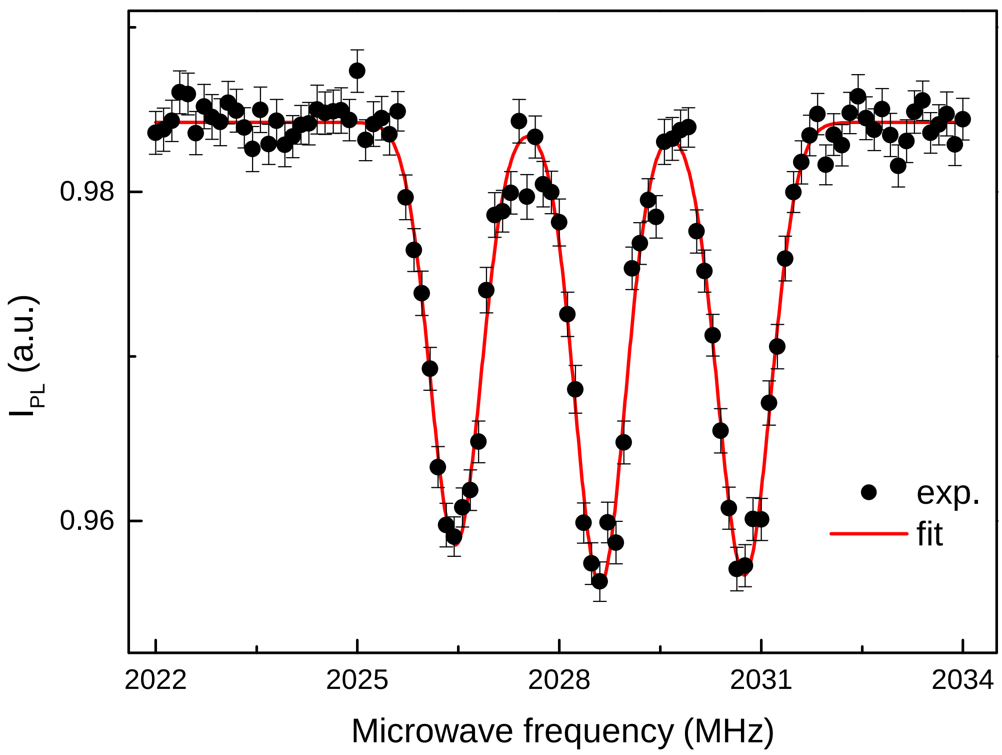

Supplementary Figure 9: **Spectrum of the electron spin of the NV center.** The distribution of δ_0, which arises from fluctuations of the static magnetic field, the microwave frequency, and the Overhauser field caused by the nuclear spin bath, can be obtained by fitting the experimental data. Error bars of the experimental data represent s.e.m.

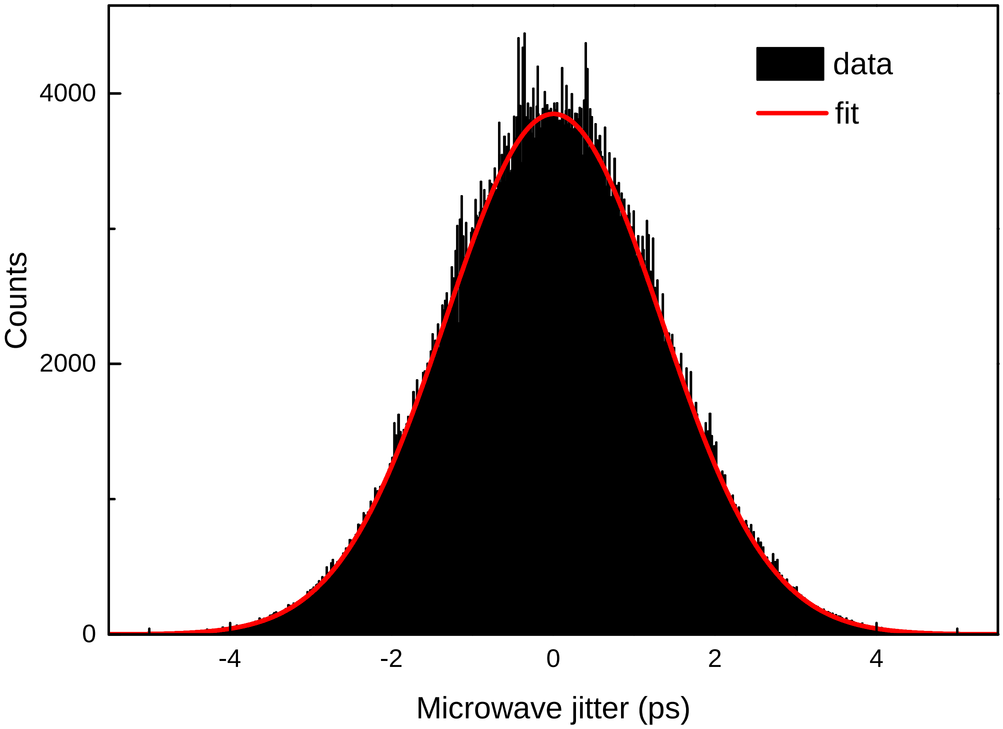

Supplementary Figure 10: **Measurement of the microwave long-term jitter.**

Supplementary Note 1: Experimental setup

An optically detected magnetic resonance (ODMR) setup combined with an atomic force microscope (AFM) is constructed to investigate the ALP-mediated interaction between an electron spin of a nitrogen-vacancy (NV) center in diamond and a fused silica half-ball lens. The schematic of the experimental setup is shown in Supplementary Fig. 1. The diamond was installed in an ODMR setup. The ODMR setup consists of optical and microwave systems. The optical system enables state initialization and readout of the electron spin via a 532 nm laser pulse. The laser pulse passed through an acousto-optic modulator (AOM, ISOMET 1250C) and an objective (Olympus, LUCPLFLN 60X, NA 0.7) before being focused on the NV center. The AOM was passed through twice by the laser pulse to preserve the longitudinal relaxation time of the NV center from laser leakage effects. When a 532 nm laser pulse is applied, phonon sideband fluorescence with wavelength of 650–800 nm can be detected to determine the state of the electron spin. The fluorescence went through the same objective and was collected by an avalanche photodiode (Perkin Elmer SPCM-AQRH-14) with a counter card. To increase the fluorescence collection efficiency, nanopillars has been fabricated on the diamond by electron beam lithography (EBL) and reactive ion etching (RIE). The microwave system enables state manipulation of the electron spin. Microwave pulses were generated by IQ modulation. The IQ baseband was provided by a 4.2 GSa/s arbitrary waveform generator (AWG, Keysight 81180A). A vector signal generator (VSG, Keysight E8267D) was used as the IQ modulator and also provided the carrier frequency. The phase and amplitude balance of the modulator were carefully calibrated with a signal analyzer (Keysight N9020A). The generated microwave pulses were amplified by a power amplifier (Mini-Circuits ZHL-16W-43-S+) and delivered by a copper microwave wire with a diameter of 20 μm to manipulate the electron spin state.

The quantum sensor to detecting spin-mass interaction is an NV center in diamond. The NV center was created by implantation of 10 keV N_2^+ ions into [100] bulk diamond and annealing for 2 hours at 800 °C in vacuum. The implantation depth is estimated to be 5-11 nm below the diamond surface[1]. Then the diamond was oxidatively etched for 4 hours at 580 °C. After oxidative etching, the depth of the NV center is estimated to be 1–7 nm[2]. Fluorescence microscopy image of NV

centers in diamond has been shown in Supplementary Fig. 2a. The NV center which is utilized in our experiment is labelled by a dashed white circle. The average separation between NV centers is about 2 μm. The second-order correlation function $g^2(\tau)$ (Supplementary Fig.2b) has been measured, where an exponential fit[3] has been applied on the experimental data and $g^2(0) < 1/2$ indicates that it is a single NV center[4].

The Hamiltonian of the NV center is

$$H = DS_z^2 + \omega_S S_z + PI_z^2 - \omega_I I_z + A_{\text{en}} S_z I_z + \sqrt{2}\omega_1 \cos(\omega t - \varphi_{\text{mw}}) S_x + \gamma B_{\text{eff}} \cos\theta S_z, \quad (1)$$

where S_x, S_y, and S_z (I_x, I_y, and I_z) are spin operators of the NV center electron spin (^{14}N nuclear spin), $D = 2\pi \times 2.87$ GHz is the zero field splitting of the electron spin, $P = -2\pi \times 4.95$ MHz is the quadrupolar splitting of the ^{14}N nuclear spin, $\omega_S = \gamma B_0$ and $\omega_I = \gamma_{\text{n}} B_0$ are Zeeman splittings of the electron and ^{14}N nuclear spins, γ and γ_{n} are the gyromagnetic ratio of the electron and ^{14}N nuclear spins, $B_0 = 300$ Gauss is the external static magnetic field, A_{en} is the hyperfine coupling constant, ω_1, ω, and φ_{mw} corresponds to the amplitude, angular frequency, and phase of the microwave pulse, $B_{\text{eff}} \cos\theta$ is the effective magnetic field along the NV symmetry axis due to the spin-mass interaction between the electron spin and the half-ball lens. In the experiment, the ^{14}N nuclear spin state keeps an unchanged mixed state

$$\rho_{^{14}\text{N}} = P_{|m_I=1\rangle} |m_I = 1\rangle\langle m_I = 1| + P_{|m_I=0\rangle} |m_I = 0\rangle\langle m_I = 0| + P_{|m_I=-1\rangle} |m_I = -1\rangle\langle m_I = -1|, \quad (2)$$

where $P_{|m_I=1\rangle}$, $P_{|m_I=0\rangle}$, and $P_{|m_I=-1\rangle}$ are populations of state $|m_I = 1\rangle$, $|m_I = 0\rangle$, and $|m_I = -1\rangle$, respectively. If the ^{14}N nuclear spin is in state $|m_I = m_k\rangle$ ($m_k = 1, 0, -1$), the reduced Hamiltonian of the electron spin is

$$H_{\text{e}}^{(m_k)} = DS_z^2 + (\omega_S + m_k A_{\text{en}}) S_z + \sqrt{2}\omega_1 \cos(\omega t - \varphi_{\text{mw}}) S_x + \gamma B_{\text{eff}} \cos\theta S_z, \quad (3)$$

The angular frequency of the microwave pulses is set to match the energy gap between $|m_S = 0\rangle$ and $|m_S = -1\rangle$. The population of state $|m_S = 1\rangle$ keeps unchanged during the experiment due to the large detuning. The Hamiltonian in the subspace composed of $|m_S = 0\rangle$ and $|m_S = -1\rangle$ can

be written as

$$H_{\mathrm{s}}^{(m_k)} = -\frac{1}{2}(D - \omega_S - m_k A_{\mathrm{en}})\sigma_z + \omega_1 \cos(\omega t - \varphi_{\mathrm{mw}})\sigma_x + \frac{1}{2}\gamma B_{\mathrm{eff}} \cos\theta \sigma_z, \tag{4}$$

where σ_x, σ_y, and σ_z are Pauli operators. Due to the state populations of the nuclear spin, the Hamiltonian can be described as

$$H_{\mathrm{s}} = -\frac{1}{2}\omega_0 \sigma_z + \omega_1 \cos(\omega t - \varphi_{\mathrm{mw}})\sigma_x + \frac{1}{2}\gamma B_{\mathrm{eff}} \cos\theta \sigma_z, \tag{5}$$

where ω_0 satisfies the distribution

$$P_{\omega_0,\mathrm{ideal}}(\omega_0) = \sum_{m_k=-1}^{1} P_{|m_I=m_k\rangle} \delta(\omega_0 - (D - \omega_S - m_k A_{\mathrm{en}})), \tag{6}$$

with $\delta(\cdot)$ being the δ function. The state evolution of the electron spin can be viewed clearly in a rotating frame. The effective Hamiltonian under rotating-wave approximation in the rotating frame is

$$H_{\mathrm{eff}} = \frac{1}{2}(\delta_0 + \gamma B_{\mathrm{eff}} \cos\theta)\sigma_z + \frac{1}{2}\omega_1[\cos(\varphi_{\mathrm{mw}})\sigma_x + \sin(\varphi_{\mathrm{mw}})\sigma_y], \tag{7}$$

where $\delta_0 = \omega - \omega_0$ is the detuning of the microwave frequency without the half-ball lens. When a microwave pulse with length $\pi/(2\omega_1)$ or π/ω_1 is applied, the evolution operator under H_{eff} approximates to that of a $\pi/2$ or π pulse. When there is no microwave pulses applied, i.e. $\omega_1 = 0$, the phase of the electron spin state accumulates with an angular frequency of $\delta_0 + \gamma B_{\mathrm{eff}} \cos\theta$. The accumulated phase of the electron spin state during the spin echo sequence is

$$\varphi_{\mathrm{tot}} = \varphi + \int_{\tau/2}^{3\tau/2} \delta_0 dt - \int_{3\tau/2}^{5\tau/2} \delta_0 dt, \tag{8}$$

where

$$\varphi = \int_{\tau/2}^{3\tau/2} \gamma B_{\mathrm{eff}} \cos\theta dt - \int_{3\tau/2}^{5\tau/2} \gamma B_{\mathrm{eff}} \cos\theta dt, \tag{9}$$

is the accumulated phase due to the spin-mass interaction. During the experiment, δ_0 is quasistatic and satisfies the distribution

$$P_{\delta_0,\mathrm{ideal}}(\delta_0) = \sum_{m_k=-1}^{1} P_{|m_I=m_k\rangle} \delta(\delta_0 - [\omega - (D - \omega_S - m_k A_{\mathrm{en}})]), \tag{10}$$

according to Supplementary Equation 6. Since δ_0 is a constant in each trail of experiment, Supplementary Equation 8 reduces to $\varphi_{\text{tot}} = \varphi$. In practice, the distribution of δ_0, $P_{\delta_0}(\delta_0)$, deviates from $P_{\delta_0,\text{ideal}}(\delta_0)$ in Supplementary Equation 10 due to the fluctuations of the static magnetic field, the microwave frequency, and the Overhauser field arisen from the nuclear spin bath. The time-dependent part of δ_0, which is described by stochastic noise with zero average, will cause decoherence of the electron spin rather than a shift of the accumulated phase when averaged over the distribution of δ_0 [5,6]. The measurement of $P_{\delta_0}(\delta_0)$ and its effect on the experimental result will be discussed in section .

The required precision for pulse timing which ensures an error $\delta\varphi$ much smaller than the measurement uncertainty of φ is discussed. According to Supplementary Equation 8, the error caused by δt is estimated as

$$\delta\varphi = \int P_{\delta_0}(\delta_0)(\delta_0 \delta t)d\delta_0, \tag{11}$$

considering the distribution $P_{\delta_0}(\delta_0)$ of δ_0. Supplementary Equation 11 reduces to

$$\delta\varphi = \langle\delta_0\rangle\delta t, \tag{12}$$

where

$$\langle\delta_0\rangle = \int P_{\delta_0}(\delta_0)\delta_0 d\delta_0, \tag{13}$$

is the mean of δ_0 over the distribution $P_{\delta_0}(\delta_0)$. Because of the symmetry of $P_{\delta_0}(\delta_0)$ (see section), $\langle\delta_0\rangle$ is very close to $\omega - (D - \omega_S)$. In the experiment, we keep the microwave angular frequency to be $\omega = D - \omega_S = 2\pi \times 2.0286$ GHz to promise that the error caused by the precision of pulse timing is negligible. Even a large detuning of $\langle\delta_0\rangle = 2\pi \times 0.1$ MHz requires $\delta t = 1.6$ ns to produce an error of $\delta\varphi = 10^{-3}$ rad, which is an order lower than the uncertainty of measured φ. The time precision in our experiment, which is 0.24 ns according to the sample rate of the AWG, is sufficient to promise that the error caused by the precision of pulse time is negligible.

The fused silica half-ball lens, which is denoted as M in the main text and hereafter, was supplied by Edmund Optics Inc. According to the datasheet, the radius of M is $250(2.5)$ μm. The surface roughness of M is estimated to be 0.01 μm by measurements with AFM. M can be

positioned near and away from the NV center by an AFM system. The AFM system is composed of a positioning system and a tuning fork. The positioning system is used to determine and change the location of M. The tuning fork is utilized to drive M to vibrate. The vibration angular frequency and amplitude can be controlled from the software of the AFM controller (Asylum Research MFP3D). The angular frequency is set to be $\omega_{\mathrm{m}} = 2\pi \times 187.29$ kHz to match a natural frequency of the tuning fork. The vibration amplitude depends on the driving voltage set from the software. By analyzing the noise spectrum of the tuning fork, a coefficient of 6.85 nm/V is obtained. The driving voltage is set to 6.00 V. Therefore, the vibration amplitude of M is estimated to be $A = 41.1(1)$ nm, where the uncertainty is estimated from the precision of the coefficient and driving voltage.

The state initialization, manipulation, readout of the electron spin, and the vibration of M, are synchronized by an arbitrary sequence generator (ASG, Hefei Quantum Precision Device Co. ASG-GT50-C). The pulse sequence is shown in Supplementary Fig. **3**. Pulse length of $\pi/2$ (π) is 59 (118) ns. The measured experimental data is $I_{\mathrm{PL}} = I_{\mathrm{S}}/I_{\mathrm{R}}$, where I_{S} and I_{R} are the photon counting corresponding to the electron spin state after the microwave sequence and that of $|m_S = 0\rangle$, respectively [7]. The pulse sequence is carried out for one million trails in each run, and the experimental result in the main text corresponds to the data of six runs. In each trail the photon counting is accumulated for 350 ns in the measurement of I_{S} or I_{R}, with an average rate of 10^5 counts/s. An oscillating electronic signal in phase with the vibration is generated due to the piezoelectric effect of quartz tuning fork. The oscillating signal is transformed into a periodic rectangular pulse train by a comparator. The ASG can be triggered by the rectangular pulse train to generate rectangular pulses, which are used to trigger or control the AWG, counter card, and the AOM. Supplementary Fig. **4** presents the recorded the microwave pulses, the oscillating electronic signal from the tuning fork and the trigger for laser. It is clear that the trigger of laser pulses, the microwave pulse sequences and the moving of the tuning fork are synchronized very well.

Supplementary Note 2: Effective magnetic field from the half-ball lens

The effective magnetic field arising from the hypothetical monopole-dipole interaction between

the electron spin and a nucleon is

$$\mathbf{B}_{\text{sp}}(\mathbf{r}) = \frac{\hbar g_{\text{s}}^{\text{N}} g_{\text{p}}^{\text{e}}}{4\pi m\gamma}(\frac{1}{\lambda r} + \frac{1}{r^2})e^{-\frac{r}{\lambda}}\mathbf{e}_r. \tag{14}$$

The effective magnetic field from the half-ball lens M can be derived by integrating $\mathbf{B}_{\text{sp}}(\mathbf{r})$ over all the nucleons in M,

$$\mathbf{B}_{\text{eff}} = \int_V \mathbf{B}_{\text{sp}}(\mathbf{r})\rho dV. \tag{15}$$

The component of $\mathbf{B}_{\text{eff}}$ perpendicular to the symmetry axis of M is zero by symmetry. Therefore, $\mathbf{B}_{\text{eff}}$ can be written as

$$\mathbf{B}_{\text{eff}} = \mathbf{e}_{r_{\text{c}}} B_{\text{eff}}, \tag{16}$$

where $\mathbf{e}_{r_{\text{c}}}$ is the unit distance vector along the symmetry axis of M. B_{eff} can be derived by integrating the component of $\mathbf{B}_{\text{sp}}(\mathbf{r})$ along the symmetry axis of M over all the nucleons in M,

$$B_{\text{eff}} = \int_V \frac{\hbar g_{\text{s}}^{\text{N}} g_{\text{p}}^{\text{e}}}{4\pi m\gamma}(\frac{1}{\lambda r} + \frac{1}{r^2})e^{-\frac{r}{\lambda}}\frac{z}{r}\rho dV, \tag{17}$$

where z is the component of $\mathbf{r}$ along the symmetry axis. The integration can be calculated in a cylindrical coordinate system. The volume element dV is written as

$$dV = ldld\phi dz, \tag{18}$$

where l is the radial distance and ϕ is the azimuth. The distance r can be described with the cylindrical coordinate as

$$r = \sqrt{z^2 + l^2}, \tag{19}$$

By substituting Supplementary Equations 18 and 19 into Supplementary Equation 17, we get

$$B_{\text{eff}} = \int_d^{d+R} dz \int_0^{\sqrt{R^2-(d+R-z)^2}} dl \int_0^{2\pi} d\phi[\frac{\hbar g_{\text{s}}^{\text{N}} g_{\text{p}}^{\text{e}}}{4\pi m\gamma}(\frac{1}{\lambda\sqrt{z^2+l^2}} + \frac{1}{z^2+l^2})e^{-\frac{\sqrt{z^2+l^2}}{\lambda}}\frac{z}{\sqrt{z^2+l^2}}\rho l]. \tag{20}$$

The integration in Supplementary Equation 20 is completed to derive B_{eff},

$$B_{\text{eff}} = \frac{\hbar g_{\text{s}}^{\text{N}} g_{\text{p}}^{\text{e}}\rho}{2m\gamma} f(\lambda, R, d), \tag{21}$$

with

$$\begin{aligned} f(\lambda, R, d) = \lambda[&\frac{R}{d+R}e^{-\frac{d}{\lambda}} - e^{-\frac{d+R}{\lambda}} + e^{-\frac{\sqrt{R^2+(d+R)^2}}{\lambda}} + \frac{\lambda\sqrt{R^2+(d+R)^2}}{(d+R)^2}e^{-\frac{\sqrt{R^2+(d+R)^2}}{\lambda}} \\ &- \frac{\lambda d}{(d+R)^2}e^{-\frac{d}{\lambda}} + \frac{\lambda^2}{(d+R)^2}e^{-\frac{\sqrt{R^2+(d+R)^2}}{\lambda}} - \frac{\lambda^2}{(d+R)^2}e^{-\frac{d}{\lambda}}]. \end{aligned} \tag{22}$$

Supplementary Note 3: Upper bound of $g_s^N g_p^e$

According to the state evolution, the final state of the electron spin after the spin echo sequence is $\cos[(\varphi_{\text{mw}} + \varphi)/2]|0\rangle + e^{i\varphi_{\text{mw}}} \sin[(\varphi_{\text{mw}} + \varphi)/2]|1\rangle$, with

$$\varphi = \int_{\tau/2}^{3\tau/2} \gamma B_{\text{eff}} \cos\theta dt - \int_{3\tau/2}^{5\tau/2} \gamma B_{\text{eff}} \cos\theta dt. \tag{23}$$

We search for the hypothetical monopole-dipole interaction, $g_s^N g_p^e$, by measuring φ. According to Supplementary Equations 21 and 23, the coupling $g_s^N g_p^e$ can be derived as

$$g_s^N g_p^e = \frac{\varphi}{h(\lambda; R, d_0, A, \theta)}, \tag{24}$$

where

$$h(\lambda; R, d_0, A, \theta) = \frac{\hbar\rho\cos\theta}{2m}[\int_{\tau/2}^{3\tau/2} f(\lambda, R, d)dt - \int_{3\tau/2}^{5\tau/2} f(\lambda, R, d)dt]. \tag{25}$$

and

$$d = d_0 + A[1 + \cos(\omega_m t)] \tag{26}$$

describes the vibration of M. The experimental result gives $\varphi = 0 \pm 0.018$ rad. Therefore, the effect of the hypothetical monopole-dipole interaction is not observed, but an upper bound of $g_s^N g_p^e$ can be set according to the uncertainties of φ and the experimental parameters such as d_0 and A.

We take systematic errors into consideration to set the upper bound of $g_s^N g_p^e$. The systematic errors are discussed in detail in section , from which it will be shown that the corrections to $g_s^N g_p^e$ due to the systematic errors are negligible. The upper bound of $g_s^N g_p^e$ is calculated as

$$\sup(g_s^N g_p^e) = \frac{\sup(\varphi)}{\min[h(\lambda; R, d_0, A, \theta)]}, \tag{27}$$

where $\sup(\varphi)$ represents the upper bound of φ, and $\min[h(\lambda; R, d_0, A, \theta)]$ is the minimum value of $h(\lambda; R, d_0, A, \theta)$. We take $\sup(\varphi) = |\varphi_{\text{sys}}| + 2\delta_\varphi$, where φ_{sys} is the contribution to the accumulated phase φ due to the systematic errors, and δ_φ is the uncertainty of the measured φ. The minimum value of $h(\lambda; R, d_0, A, \theta)$ is numerically calculated with the parameters R, d_0, A, and θ taken within the uncertainty ranges. The derived upper bound of $g_s^N g_p^e$ is shown as the red solid line in Fig. 4 in the main text.

The result can be further improved. Since the sensitivity for detecting the effective magnetic field is limited by the coherence time of the electron spin, a method to improve the bound is to prolong the coherence time. The coherence time of the near surface NV center in our experiment is prolonged from $T_2^* = 0.67$ μs to $T_2 = 8.3$ μs with a spin echo sequence. By applying multi-pulse dynamical decoupling sequences such as a CPMG sequence, the coherence time can be further prolonged. Recently, a coherence time of $T_{2,\text{CPMG1024}} = 1280$ μs is reported for near-surface NV centers with a CPMG-1024 sequence applied [8]. The vibration of M can be controlled to match the CPMG sequence. For example, M can be driven to vibrate with an angular frequency of $\omega_\text{m} = K\pi/T_{2,\text{CPMG1024}} = 2\pi \times 400$ kHz, where $K = 1024$ is the number of π pulses. The CPMG-1024 sequence is synchronized with the vibration of M such that the π pulses are applied only when M is passing through the equilibrium point of the vibration. In this case, Supplementary Equation 24 still holds as long as Supplementary Equation 25 is replaced by

$$h(\lambda; R, d_0, A, \theta) = \frac{\hbar K \rho \cos\theta}{4m}\left[\int_{\tau/2}^{3\tau/2} f(\lambda, R, d)dt - \int_{3\tau/2}^{5\tau/2} f(\lambda, R, d)dt\right]. \tag{28}$$

According to Supplementary Equation 24, strategies to reduce the uncertainty of φ and those to maximize $h(\lambda; R, d_0, A, \theta)$ can be taken to improve the result. A straightforward way to increase $h(\lambda; R, d_0, A, \theta)$ is to use materials with higher ρ, such as Bi4Ge3O12 (BGO), as the nucleon source. The number density of nucleons in BGO is $\rho = 4.29 \times 10^{30}$ m^{-3}. Another method is to optimize the geometry parameters such as d_0 and A to increase $h(\lambda; R, d_0, A, \theta)$. The distance d_0 can be set to about 100 nm, and the amplitude of the vibration can be improved to $A = 400$ nm. To reduce the uncertainty of φ, we can improve the detection efficiency of the photoluminescence and increase the number of experiment scans. The detection efficiency can be improved to achieve a photoluminescence rate of 1.7×10^6 counts/s [9], which is 17 times larger than that in the current experiment. Considering these improvements, the estimated available upper bound of $g_\text{s}^\text{N} g_\text{p}^\text{e}$ is shown as the red dashed line in Fig. 4 in the main text, which is about 3 orders of magnitude more stringent than that from the current experiment result. The systematic errors caused by the diamagnetism of the half-ball lens and the tuning fork are estimated in this case by simulation. The simulation shows that the estimated improved upper bound of $g_\text{s}^\text{N} g_\text{p}^\text{e}$ has not reached the limit set by

intrinsic systematic errors caused by the diamagnetism.

Supplementary Note 4: Experimental results and error analysis

Experimental results and statistical error in φ. We carried out six separated runs of the measurement with and without mass M, which are shown in Supplementary Fig. 5. Each run contains one million trails. The experimental sequences (shown in Supplementary Fig. 3) of each run are the same. Photon counting is accumulated for 350 ns for measurement of I_{S} or I_{R} in each trail, with an average rate of 10^5 counts/s. In the main text, the result is the sum of the six runs. Phase differences between experiments with and without the mass can be derived from the experimental data. Supplementary Fig. 6 includes the obtained phase differences. Our results show that the reproducibility of the measurement is good. From the phase difference considering the results of all the six runs, the statistical error in φ is obtained to be $\delta_{\varphi} = 0.018$ rad.

Diamagnetism of the half-ball lens. A systematic error in measurement of φ is due to the magnetic field $\mathbf{B}_{\text{M, diam}}$ on the NV center caused by the diamagnetism of the half-ball lens. Because of the external magnetic field $\mathbf{B}_0$, there is magnetic dipole moment in the diamagnetic half-ball lens, which produces the magnetic field $\mathbf{B}_{\text{M, diam}}$ on the NV center, as is shown in Supplementary Fig. 7. The magnetic field $\mathbf{B}_{\text{M, diam}}$ is calculated as follows.

The magnetization $\mathbf{M}$ of the half-ball lens in the magnetic field $\mathbf{B}_0$ can be written as

$$\mathbf{M} = \frac{\chi}{\mu_0}\mathbf{B}_0, \tag{29}$$

where $\chi = -11.28 \times 10^{-6}$ is the magnetic susceptibility of the half-ball lens, and μ_0 is the vacuum permeability. The magnetic dipole moment $d\mathbf{m}$ of a volume element dV at position $\mathbf{r}$ in the half-ball lens is

$$d\mathbf{m} = \mathbf{M}dV. \tag{30}$$

Such a magnetic dipole moment $d\mathbf{m}$ will cause a magnetic field $d\mathbf{B}_{\text{M, diam}}$ on the NV center

$$d\mathbf{B}_{\text{M, diam}} = \frac{\mu_0}{4\pi}\{\frac{3(-\mathbf{r})[d\mathbf{m}\cdot(-\mathbf{r})]}{r^5} - \frac{d\mathbf{m}}{r^3}\}, \tag{31}$$

where $r = |\mathbf{r}|$ is the distance between the NV center and the volume element dV. The magnetic field $\mathbf{B}_{\text{M, diam}}$ on the NV center due to the diamagnetism of the half-ball lens can be obtained by integrating $d\mathbf{B}_{\text{M, diam}}$ over the whole volume of the half-ball lens

$$\mathbf{B}_{\text{M, diam}} = \int_V d\mathbf{B}_{\text{M, diam}}, \tag{32}$$

which gives

$$\mathbf{B}_{\text{M, diam}} = \int_V \frac{\chi}{4\pi}[\frac{3\mathbf{r}(\mathbf{B}_0 \cdot \mathbf{r})}{r^5} - \frac{\mathbf{B}_0}{r^3}]dV, \tag{33}$$

We calculate the components of $\mathbf{B}_{\text{M, diam}}$ parallel and perpendicular to the symmetry axis of the NV center. $\mathbf{B}_{\text{M, diam}}$ is written as

$$\mathbf{B}_{\text{M, diam}} = \mathbf{e}_{\|} B_{\text{M, diam},\|} + \mathbf{e}_{\perp 1} B_{\text{M, diam},\perp 1} + \mathbf{e}_{\perp 2} B_{\text{M, diam},\perp 2}, \tag{34}$$

where $\mathbf{e}_{\|} = (\mathbf{e}_x + \mathbf{e}_y + \mathbf{e}_z)/\sqrt{3}$, $\mathbf{e}_{\perp 1} = (\mathbf{e}_x - \mathbf{e}_y)/\sqrt{2}$, and $\mathbf{e}_{\perp 2} = (\mathbf{e}_x + \mathbf{e}_y - 2\mathbf{e}_z)/\sqrt{6}$ are unit vectors parallel ($\mathbf{e}_{\|}$) and perpendicular ($\mathbf{e}_{\perp 1}$ and $\mathbf{e}_{\perp 2}$) to the NV symmetry axis. The static magnetic field, $\mathbf{B}_0 = \mathbf{e}_{\|} B_0$, is along the NV symmetry axis with a magnitude $B_0 = 300$ Gauss. The components of $\mathbf{B}_{\text{M, diam}}$ is derived as

$$B_{\text{M, diam},\|} = \frac{\chi B_0}{4\pi} \int_V [\frac{3(\mathbf{e}_{\|} \cdot \mathbf{r})^2}{r^5} - \frac{1}{r^3}]dV, \tag{35}$$

$$B_{\text{M, diam},\perp 1} = \frac{\chi B_0}{4\pi} \int_V \frac{3(\mathbf{e}_{\perp 1} \cdot \mathbf{r})(\mathbf{e}_{\|} \cdot \mathbf{r})}{r^5} dV, \tag{36}$$

$$B_{\text{M, diam},\perp 2} = \frac{\chi B_0}{4\pi} \int_V \frac{3(\mathbf{e}_{\perp 2} \cdot \mathbf{r})(\mathbf{e}_{\|} \cdot \mathbf{r})}{r^5} dV. \tag{37}$$

In the following, we firstly calculate $\mathbf{B}_{\text{M, diam}}$ and its effect on the accumulated phase of the state of the NV center electron spin in the case that the NV center locates exactly under the center of the half-ball lens, and then consider the case that the NV center is not exactly positioned under the center of the half-ball lens. The results show that the effect of $\mathbf{B}_{\text{M, diam}}$ on the accumulated phase of the NV center electron spin state is negligible in both cases.

If the NV center locates exactly under the center of the half-ball lens, the integrations in Supplementary Equations 35, 36, and 37 can be calculated in the cylindrical coordinate system as

follows

$$B_{\text{M, diam},\parallel} = \frac{\chi B_0}{4\pi}\int_d^{d+R} dz \int_0^{\sqrt{R^2-(d+R-z)^2}} dl \int_0^{2\pi} d\phi \frac{2l^2[z\cos(\phi)+z\sin(\phi)+(l/2)\sin(2\phi)]}{(l^2+z^2)^{5/2}}, \tag{38}$$

$$B_{\text{M, diam},\perp 1} = \frac{\chi B_0}{4\pi}\int_d^{d+R} dz \int_0^{\sqrt{R^2-(d+R-z)^2}} dl \int_0^{2\pi} d\phi \frac{\sqrt{3}l^2[z\cos(\phi)-z\sin(\phi)+l\cos(2\phi)]}{\sqrt{2}(l^2+z^2)^{5/2}}, \tag{39}$$

$$B_{\text{M, diam},\perp 2} = \frac{\chi B_0}{4\pi}\int_d^{d+R} dz \int_0^{\sqrt{R^2-(d+R-z)^2}} dl \int_0^{2\pi} d\phi \frac{l[l^2-2z^2-zl\cos(\phi)-zl\sin(\phi)+l^2\sin(2\phi)]}{\sqrt{2}(l^2+z^2)^{5/2}}. \tag{40}$$

After completing the integrations in Supplementary Equations 38, 39, and 40,

$$B_{\text{M, diam},\parallel} = 0, \tag{41}$$

$$B_{\text{M, diam},\perp 1} = 0, \tag{42}$$

$$B_{\text{M, diam},\perp 2} = \frac{\chi B_0}{2\sqrt{2}}\{\frac{-2[(d+R)^2+R^2]^{3/2}+2d^3}{3(d+R)^3} - \frac{2d^2}{(d+R)^2} + \frac{\sqrt{(d+R)^2+R^2}+2d}{d+R} - \frac{d+R}{\sqrt{(d+R)^2+R^2}}\}. \tag{43}$$

The magnetic field on the NV center caused by the diamagnetism of the half-ball lens, $\mathbf{B}_{\text{M, diam}} = \mathbf{e}_{\perp 2}B_{\text{M, diam},\perp 2}$, is perpendicular to the NV symmetry axis. The magnetic field $\mathbf{B}_{\text{M, diam}}$ will cause an energy shift $\sim(\gamma B_{\text{M, diam},\perp 2})^2/\omega_0$ of the NV center electron spin, where $\omega_0 = 2\pi \times 2.0286$ GHz is the energy gap of the electron spin. The distance between the bottom of the half-ball lens and the NV center is $d = d_0 + A[1+\cos(\omega_{\text{m}}t)]$, with $d_0 = 0.5(1)$ μm and the vibration amplitude $A = 41.1(1)$ nm. During the vibration of the half-ball lens, $B_{\text{M, diam},\perp 2}$ is in the range from 1.3995×10^{-3} Gauss to 1.4010×10^{-3} Gauss, corresponding to the energy shift in the range from $2\pi \times 7.585 \times 10^{-3}$ Hz to $2\pi \times 7.601 \times 10^{-3}$ Hz. The contribution of $\mathbf{B}_{\text{M, diam}}$ to the accumulated phase of the electron spin state, estimated by $\delta\varphi_M = \int_{\tau/2}^{3\tau/2}(\gamma B_{\text{M, diam},\perp 2})^2/\omega_0 dt - \int_{3\tau/2}^{5\tau/2}(\gamma B_{\text{M, diam},\perp 2})^2/\omega_0 dt$, is 1.7×10^{-10} rad with the waiting time $\tau = 2.67$ μs.

Because the NV center is not exactly positioned under the center of the mass, the misalignment between the NV center and the center of the half-ball lens is estimated to be taken into consi-

deration. The misalignment can be estimated from the CCD image shown in Supplementary Fig. 8. The light spot in the image shows the location of the NV center, which is focused in the laser light. The rings in the image are Newton rings due to the half-ball lens. The location of the center of the half-ball lens in the horizontal plane is determined by fitting the position of Newton rings. According to the locations of the NV center and the center of the half-ball lens, the misalignment is estimated to be $0.7(8)$ μm. Considering the misalignment, $\mathbf{B}_{\text{M, diam}}$ is numerically calculated according to Supplementary Equations 35, 36, and 37. As an example, when the misalignment is taken to be 0.7 μm, $B_{\text{M, diam,}\parallel}$ is in the range from 8.4096×10^{-6} Gauss to 8.4210×10^{-6} Gauss, and $B_{\text{M, diam,}\perp} = \sqrt{B^2_{\text{M, diam,}\perp1} + B^2_{\text{M, diam,}\perp2}}$ is in the range from 1.3964×10^{-3} Gauss to 1.3979×10^{-3} Gauss. The contribution of $\mathbf{B}_{\text{M, diam}}$ to the accumulated phase of the electron spin state is calculated by $\delta\varphi_{\text{M}} = \int_{\tau/2}^{3\tau/2}[\gamma B_{\text{M, diam,}\parallel} + (\gamma B_{\text{M, diam,}\perp})^2/\omega_0]dt - \int_{3\tau/2}^{5\tau/2}[\gamma B_{\text{M, diam,}\parallel} + (\gamma B_{\text{M, diam,}\perp})^2/\omega_0]dt$. Considering the misalignment and its uncertainty, $\delta\varphi_M$ is calculated to be $3(3) \times 10^{-7}$ rad, which is much smaller than the uncertainty (0.018 rad) in measurement of φ. The correction to $g_{\text{s}}^{\text{N}}g_{\text{p}}^{\text{e}}$ caused by $\mathbf{B}_{\text{M, diam}}$ is calculated and taken into consideration when setting the upper bound on $g_{\text{s}}^{\text{N}}g_{\text{p}}^{\text{e}}$ in the main text. As an example, such a correction at the force range $\lambda = 20$ μm is $5(5) \times 10^{-20}$.

Diamagnetism of the tuning fork. The numerical calculation of the magnetic field caused by the diamagnetism of the tuning fork and its effect on the accumulated phase of the NV center electron spin state is similar to that for the half-ball lens. Each prong of the tuning fork can be considered as a 2.5 mm $\times$ 0.22 mm $\times$ 0.46 mm cube. The distance between the undersurface of the tuning fork prong and the NV center is larger than $R = 250$ μm. The magnetic susceptibility of the tuning fork is -11.28×10^{-6}. When vibrating, a time-varying magnetic field $\mathbf{B}_{\text{TF,diam}}$ can be produced by the diamagnetism of the tuning fork. The component $B_{\text{TF,diam,}\parallel}$ of $\mathbf{B}_{\text{TF,diam}}$ parallel to the NV symmetry axis is estimated to be in the range from 4.7967×10^{-5} Gauss to 4.7975×10^{-5} Gauss, and the component $B_{\text{TF,diam,}\perp}$ perpendicular to the NV symmetry axis is in the range from 1.8424×10^{-4} Gauss to 1.8433×10^{-4} Gauss. The contribution of $\mathbf{B}_{\text{TF,diam}}$ to the accumulated phase of the NV center electron spin state is calculated to be $\delta\varphi_{\text{TF}} = 2.5(2) \times 10^{-7}$ rad, where the uncertainty 0.2×10^{-7} rad in $\delta\varphi_{\text{TF}}$ is estimated according to the estimated uncertainty in the horizontal position of the tuning fork. The correction to $g_{\text{s}}^{\text{N}}g_{\text{p}}^{\text{e}}$ caused by $\mathbf{B}_{\text{TF,diam}}$, which is $3.8(3) \times 10^{-20}$ at $\lambda = 20$ μm

as an example, is taken into consideration when setting the upper bound on $g_{\rm s}^{\rm N}g_{\rm p}^{\rm e}$ in the main text.

Fluctuations of the static magnetic field, microwave frequency, and Overhauser field due to the nuclear spin bath. The distribution of δ_0, $P_{\delta_0}(\delta_0)$, is obtained by measuring the spectrum of the NV center electron spin with a low microwave amplitude. Supplementary Fig. 9 shows the experimental spectrum. The data can be fitted with $I_{\rm PL} = I_0 + a\sum_{m_k=-1}^{1} P_{|m_I=m_k\rangle}/(\sqrt{2\pi}\sigma_\nu)\exp\{-[\nu-(\nu_0 - m_k A_{\rm en}/2\pi)]^2/(2\sigma_\nu^2)\}$, where ν is the microwave frequency in measurement of the spectrum and $\nu_0 - m_k A_{\rm en}/2\pi$ denotes the resonant frequency with the ^{14}N nuclear spin in state $|m_I = m_k\rangle$. From fitting, $P_{|m_I=m_k\rangle}$ is estimated with an uncertainty of 0.01 to be $P_{|m_I=-1\rangle} = 0.32$, $P_{|m_I=0\rangle} = 0.34$, and $P_{|m_I=1\rangle} = 0.34$. The values of other parameters are $\sigma_\nu = 0.38(1)$ MHz, $A_{\rm en} = -2\pi \times 2.15(1)$ MHz, and $\nu_0 = 2028.60(1)$ MHz. In the experiment to detect the spin-mass interaction, the microwave frequency is set to ν_0. The distribution of δ_0 is $P_{\delta_0}(\delta_0) = \sum_{m_k=-1}^{1} P_{|m_I=m_k\rangle}/(\sqrt{2\pi}\sigma_{\delta_0})\exp[-(\delta_0+m_k A_{\rm en})^2/(2\sigma_{\delta_0}^2)]$, with $\sigma_{\delta_0} = 2\pi\sigma_\nu$. Compared to $P_{\delta_0,\rm ideal}(\delta_0)$, the δ functions in $P_{\delta_0,\rm ideal}(\delta_0)$ are replaced by Gaussian functions due to the fluctuations of the static magnetic field, microwave frequency, and Overhauser field arisen from the nuclear spin bath, which cause the dephasing of the electron spin. The value of the standard deviation σ_{δ_0} of the Gaussian functions coincides with the dephasing time $T_2^* = 0.67(4)$ μs. The state evolution of the NV center electron spin under the spin echo sequence is simulated with the distribution $P_{\delta_0}(\delta_0)$ of δ_0 taken into consideration. The simulation results show that the distribution $P_{\delta_0}(\delta_0)$ of δ_0 can cause a phase shift of $-3.0\times10^{-9} \pm 8.9\times10^{-15}$ rad to the accumulated phase of the electron spin state in both cases without and with the half-ball lens. Since only the difference of the accumulated phases with and without the half-ball lens is related to the phase φ for derivation of $g_{\rm s}^{\rm N}g_{\rm p}^{\rm e}$, the phase shift to φ caused by the distribution $P_{\delta_0}(\delta_0)$ is $0 \pm 1.3\times10^{-14}$ rad, corresponding to a correction of $0 \pm 1.9\times10^{-27}$ to $g_{\rm s}^{\rm N}g_{\rm p}^{\rm e}$ at $\lambda = 20\mu$ m. The correction to $g_{\rm s}^{\rm N}g_{\rm p}^{\rm e}$ caused by the distribution $P_{\delta_0}(\delta_0)$ is taken into consideration when setting the upper bound on $g_{\rm s}^{\rm N}g_{\rm p}^{\rm e}$ in the main text.

Microwave jitter. The measurement of the microwave long-term jitter is shown in Supplementary Fig. 10. The data can be fit by a Gaussian distribution, with a standard deviation of 1.3 ps. The microwave jitter can cause instability of microwave phase. To estimate the effect of the microwave

jitter on experimental data, we simulate the state evolution of the NV center electron spin taking the microwave jitter into consideration. The calculation shows that the microwave jitter can contribute a shift of $3.5\times10^{-5}\pm7.6\times10^{-15}$ rad to the accumulated phase of the NV center electron spin state, both in the cases without and with the half-ball lens. Since only the difference of the accumulated phases with and without the half-ball lens is related to the phase for derivation of $g_{\mathrm{s}}^{\mathrm{N}}g_{\mathrm{p}}^{\mathrm{e}}$, the contribution of the microwave jitter to the interested phase is $0\pm1.1\times10^{-14}$ rad, corresponding to a correction of $0\pm1.7\times10^{-27}$ to $g_{\mathrm{s}}^{\mathrm{N}}g_{\mathrm{p}}^{\mathrm{e}}$ at $\lambda=20\mu$ m. The correction of the microwave jitter to $g_{\mathrm{s}}^{\mathrm{N}}g_{\mathrm{p}}^{\mathrm{e}}$ is taken into consideration when setting the upper bound on $g_{\mathrm{s}}^{\mathrm{N}}g_{\mathrm{p}}^{\mathrm{e}}$ in the main text.

Uncertainty in d_0. According to Supplementary Equation 24, the uncertainty in d_0 will cause a correction to $g_{\mathrm{s}}^{\mathrm{N}}g_{\mathrm{p}}^{\mathrm{e}}$. In the experiment, d_0 is measured to be $0.5(1)$ μm. To estimate the correction of the uncertainty in d_0 to $g_{\mathrm{s}}^{\mathrm{N}}g_{\mathrm{p}}^{\mathrm{e}}$, we randomly take 1000 samples for d_0 which satisfies a Gaussian distribution $P_{d_0}(d_{0,i})=\frac{1}{\sqrt{2\pi}\sigma_{d_0}}\exp[-\frac{(d_{0,i}-\mu_{d_0})^2}{2\sigma_{d_0}^2}]$, where $\mu_{d_0}=0.5$ μm and $\sigma_{d_0}=0.1$ μm are the value and uncertainty of measured d_0. For each $d_{0,i}$, $g_{\mathrm{s}}^{\mathrm{N}}g_{\mathrm{p}}^{\mathrm{e}}|_{d_0=d_{0,i}}$ is calculated according to Supplementary Equation 24. The mean $\mu(g_{\mathrm{s}}^{\mathrm{N}}g_{\mathrm{p}}^{\mathrm{e}})$ and standard deviation $\sigma(g_{\mathrm{s}}^{\mathrm{N}}g_{\mathrm{p}}^{\mathrm{e}})$ of $g_{\mathrm{s}}^{\mathrm{N}}g_{\mathrm{p}}^{\mathrm{e}}|_{d_0=d_{0,i}}$ are calculated. The correction to $g_{\mathrm{s}}^{\mathrm{N}}g_{\mathrm{p}}^{\mathrm{e}}$, $\mu(g_{\mathrm{s}}^{\mathrm{N}}g_{\mathrm{p}}^{\mathrm{e}})-g_{\mathrm{s}}^{\mathrm{N}}g_{\mathrm{p}}^{\mathrm{e}}|_{d_0=\mu_{d_0}}\pm\sigma(g_{\mathrm{s}}^{\mathrm{N}}g_{\mathrm{p}}^{\mathrm{e}})$, is $(0.1\pm3.0)\times10^{-17}$ at $\lambda=20$ μm.

Uncertainty in A. The vibration amplitude is measured to be $A=41.1(1)$ nm. The calculation of the correction of the uncertainty in A to $g_{\mathrm{s}}^{\mathrm{N}}g_{\mathrm{p}}^{\mathrm{e}}$ is similar to that for the uncertainty in d_0. The correction is $(0.0\pm1.3)\times10^{-17}$ at $\lambda=20$ μm.

Uncertainty in R. The radius of the half-ball lens is $R=250(2.5)$ μm according to the datasheet. The calculation of the correction of the uncertainty in R to $g_{\mathrm{s}}^{\mathrm{N}}g_{\mathrm{p}}^{\mathrm{e}}$ is similar to that for the uncertainty in d_0. The correction is $(0.1\pm3.7)\times10^{-18}$ at $\lambda=20$ μm.

Uncertainty in θ. The uncertainty in θ is estimated to be 3 degree according to the miscut for the crystallographic orientation of the diamond. The calculation of the correction of the uncertainty in θ to $g_{\mathrm{s}}^{\mathrm{N}}g_{\mathrm{p}}^{\mathrm{e}}$ is similar to that for the uncertainty in d_0. The correction is $(0.4\pm4.2)\times10^{-16}$ at

$\lambda = 20\ \mu$m.

Supplementary References

1. Ziegler, J. F., Ziegler, M. D. and Biersack, J. P. SRIM – The stopping and range of ions in matter (2010). *Nucl. Instrum. Methods Phys. Res. Sect. B: Beam Interact. Mater. Atoms* **268**, 1818-1823 (2010). 12

2. Wang, J., Zhang, W., Zhang, J., You, J., Li, Y., Guo, G., Feng, F., Song, X., Lou, L., Zhu, W. and Wang, G. Coherence times of precise depth controlled NV centers in diamond. *Nanoscale* **8**, 5780-5785 (2016). 12

3. Kitson, S. C. *et al.* Intensity fluctuation spectroscopy of small numbers of dye molecules in a microcavity. *Phys. Rev. A* **58**, 620 (1998). 13

4. Brouri R. *et al.* Photon antibunching in the fluorescence of individual color centers in diamond. *Opt. Lett.* **25**, 1294 (2000). 13

5. Kofman, A. G. and Kurizki G. Unified theory of dynamically suppressed qubit decoherence in thermal baths. *Phys. Rev. Lett.* **93**, 130406 (2004). 15

6. Álvarez G. A. and Suter D. Measuring the spectrum of colored noise by dynamical decoupling. *Phys. Rev. Lett.* **107**, 230501 (2011). 15

7. Doherty, M. W. *et al.* The nitrogen-vacancy colour centre in diamond. *Phys. Rep.* **528**, 1-45 (2013). 16

8. Myers, B. A., Ariyaratne, A. and Jayich, A. C. B. Double-quantum spin-relaxation limits to coherence of near-surface nitrogen-vacancy centers. *Phys. Rev. Lett.* **118**, 197201 (2017). 19

9. Momenzadeh, S. A. *et al.* Nanoengineered diamond waveguide as a robust bright platform for nanomagnetometry using shallow nitrogen vacancy centers. *Nano Lett.* **15**, 165-169 (2014). 19
